# Impact of Technological Immersion and Sensorimotor Engagement on Performance and Brain Plasticity in Short-Term Second Language Vocabulary Training

**DOI:** 10.1162/NOL.a.238

**Published:** 2026-03-27

**Authors:** Theodor Rumetshofer, Lara Langensee, Ping Li, Jiayan Zhao, Alexander Klippel, Linda Wennberg, Markus Nilsson, Pia C. Sundgren, Marianne Gullberg, Johan Mårtensson

**Affiliations:** Department of Clinical Sciences Lund, Division of Logopedics, Phoniatrics and Audiology, Lund University, Lund, Sweden; Department of Chinese and Bilingual Studies, Faculty of Humanities, The Hong Kong Polytechnic University, Hong Kong, China; Laboratory of Geo-information Science and Remote Sensing, Wageningen University & Research, Wageningen, The Netherlands; Cultural Geography Research Group (GEO) and WANDER XR Experience Lab, Wageningen University and Research, Wageningen, The Netherlands; Department of Medical Imaging and Physiology, Skåne University Hospital, Lund, Sweden; Diagnostic Radiology, Department of Clinical Sciences Lund, Lund University, Sweden; Lund University Bioimaging Centre, Lund University, Lund, Sweden; Centre for Languages and Literature, Lund University, Lund, Sweden; Lund University Humanities Lab, Lund University, Lund, Sweden

**Keywords:** immersion, second language learning, sensorimotor engagement, ultra-high field magnetic resonance imaging (MRI), virtual reality, voxel-based morphometry

## Abstract

Classroom-based language learning has typically taken place in relatively static body positions, but research suggests that embodied learning through sensorimotor engagement and technical immersion, using virtual realities, can significantly enhance learning outcomes. Recent research has linked differences in the learning context to different cortical structures within the language learning network. In this study, we investigated the effect of technical immersion and sensorimotor engagement on performance in behavior and gray matter volume in the brain after a single 20-min language learning task. We tested two learning environments: a low-embodied desktop-based virtual environment (dVE) using a computer screen and a high-embodied immersive virtual reality (iVR) environment using a head-mounted display, as well as a no training group. We assessed morphological brain changes using magnetic resonance imaging at 7 Tesla before and after training. Participants with less sensorimotor engagement, compared to those with high, performed significantly better and showed higher gray matter volume in the left angular gyrus, a key hub region for vocabulary training within the language network, as well as in the left middle temporal gyrus, a region associated with lexical semantic processing. However, we could not identify a difference between the dVE and iVR groups. Our results suggest that both virtual platforms, although different in the level of immersion and whole-body involvement, rely on similar cortical structures within the language learning network. Furthermore, sensorimotor engagement might have a stronger influence on performance and related brain changes than the learning context itself.

## INTRODUCTION

Virtual reality (VR) technology shows potential as an experimental tool in language sciences (Peeters, 2019) as it allows for embodied language learning experiences with high ecological validity in controlled experimental settings (Li & Lan, 2022; Li et al., 2020; Peeters, 2019). Compared to first language (L1) learning, which is a highly embodied learning experience (Li et al., 2020), adult second language (L2) learning in the West mostly takes place in relatively static positions, for example, on mobile phones or in classrooms. VR technology has the potential to create enriched perceptual learning environments with a high level of immersion and sensorimotor interaction to enhance L2 learning (Li & Jeong, 2020; Peeters, 2019). The interplay of memory, body movement, and immersion can be summarized in the theoretical perspective of embodied cognition (Barsalou, 2015).

When children learn a new language, manual motor behavior and bodily actions are important predictors for learning success (Adams, 2016; Schroer & Yu, 2023; West & Iverson, 2017). Such embodied actions are also highly relevant for L2 learning in adults. For example, the production of co-speech gestures has been found to improve vocabulary learning, conceivably by adding more depth through sensorimotor activation during encoding (see Gullberg, 2024, for an overview). Also, encoding with practical actions, defined as moving or using objects during memorization, has been shown to lead to better learning outcomes compared to gestures, defined as actions without moving the object (Johnson-Glenberg et al., 2014; Ratcliffe et al., 2021).

Beside sensorimotor engagement, immersion is another main construct in VR-supported educational learning (Johnson-Glenberg, 2018). Immersion refers to the use of technology, such as visual displays, to evoke a strong sense of being deeply engaged or present in an environment. The degree of technological immersion depends on how effectively the system can produce an inclusive, extensive, and vivid representation of reality (Slater & Wilbur, 1997). When comparing two technologies or setups, for example, a desktop-based virtual environment (dVE) using a computer screen and an immersive virtual reality (iVR) setup using a head-mounted display (HMD), the latter involves a higher level of technical immersion and allows for whole-body interactions with the environment. The 360° view of iVR can shut out the physical reality more effectively compared to a computer screen and can provide a more inclusive and encompassing illusion (Zhao, LaFemina, et al., 2020). Additionally, 3D tracking of real-world user movements allows for interactions in the virtual space and creates a whole-body and vivid experience. In contrast, movements and interactions in dVE are controlled and executed by keyboard, mouse, or joystick movements, and the virtual world does not adapt to head or body movements (Zhao, Sensibaugh, et al., 2020). Multiple studies have shown learning benefits from high-embodied, technical iVR setups compared to non-virtual, non-immersive learning settings in L2 learning (Lan et al., 2015; Legault, Zhao, et al., 2019; Macedonia et al., 2020; Ratcliffe & Tokarchuk, 2021).

Higher levels of technological immersion and interaction through sensorimotor engagement benefits language learning performance, but does it also impact local brain structure, and if so, are differences related to the level of immersion and level of interaction? Only a few studies on language learning, using immersive technologies, have included neuroimaging techniques, for example, magnetic resonance imaging (MRI). In a resting-state functional MRI study, children showed an increase in functional connectivity between Broca’s and Wernicke’s area after 12 weeks of using an online English learning game (Hong et al., 2017). However, this result was not compared to a control group. In another study in an adult sample testing two different learning setups, picture–word associations with a dVE, cortical thickness, and gray matter volume changes were found in the left language control network in both groups (Legault, Fang, et al., 2019). Interestingly, performance showed a positive correlation with cortical thickness in the right inferior parietal lobe (IPL) for the low-embodied dVE and a negative correlation for the non-embodied picture–word training. Additionally, the latter shows a positive correlation in the right inferior frontal gyrus (IFG) with performance in the late training phase. These results might indicate structural differences due to learning context. The authors argued that the right language control network seems to be more sensitive to the learning context compared to the left hemisphere.

In a review on structural brain changes related to bilingualism, changes in the left IPL and left IFG seem to reflect L2 proficiency (Legault, Fang, et al., 2019; Stein et al., 2014). However, changes in those areas seem to be independent from a learning environment (i.e., naturalistic exposure and classroom setting), although variations might be associated to the age of acquisition of L2. The bilateral IPL, which comprises the supramarginal gyrus and the angular gyrus, does not only reflect learning outcome but is also associated with phonological storage, working memory, embodied cognition, and immersive learning (Li & Lan, 2022; Li et al., 2020). In general, clear lateralization effects within the L2 learning context, as compared with L1 learning, remain under fierce debate (Qi & Legault, 2020).

Synthesizing the sparse available neuroimaging findings on embodied L2 learning using immersion technologies and sensorimotor engagement, evidence may suggest a learning environment-dependent brain plasticity. However, current language studies on VR-supported language learning differ vastly in design and comparability. Therefore, in this study, we examine neural correlates of two virtual learning environments, in particular dVE and iVR, which have been shown to lead to superior learning outcomes compared to non-virtual and non-immersive learning methods (Lan et al., 2015; Legault, Fang, et al., 2019; Legault, Zhao, et al., 2019). However, a direct systematic comparison of low-embodied dVE and high-embodied iVR has not been done before. Such a comparison, by including MRI, allows us to investigate the effect of technological immersion and sensorimotor engagement on performance and potential structural brain changes. Additionally, to investigate individual differences, in particular the effect of sensorimotor engagement, we measure how often a virtual object was manipulated (e.g., moved or rotated) during learning, which is a new method for this type of study (Li et al., 2020).

Compared to non-linguistic training studies where rapid and transient brain changes have already been found, linguistic experiments normally lasted from weeks to months of training. However, an effect on structural or functional brain changes might depend on the intensity and quality of the language learning experience (Li et al., 2014). Virtual L2 learning environments have the ability to increase the intensity of the training and experience due to high embodied training (Smith, 2019).

To investigate the potential of VR-supported learning, both training groups (dVE and iVR) perform a single 20-min language learning task while a third group with no training watched a movie of a nature walk. The short vocabulary training task experienced by the dVE and iVR groups was based on evidence that even brief sessions of language learning can lead to changes in the brain (Gullberg et al., 2010; Hofstetter et al., 2017; Kwok et al., 2011; Veroude et al., 2010).

To detect potential morphological changes in the brain, all groups underwent a pre- and post-intervention ultra-high field brain MRI. We used a voxel-based morphometry (VBM) analysis to examine rapid changes in the gray matter volume. VBM is a widely used and well-known method to measure the impact of learning on gray matter volume (Johansen-Berg et al., 2012; Lövdén et al., 2013; Zatorre et al., 2012). Multiple VBM studies have shown differences in gray matter volume even after a short period of training (between 10 min and 2 hr) in vocabulary learning (Kwok et al., 2011), motor skill acquisition (Olivo et al., 2022; Taubert et al., 2016), or image viewing (Månsson et al., 2020; Naegel et al., 2017).

We hypothesize (a) that there are differences in structural brain changes between participants in a low-embodied (dVE) as opposed to high-embodied (iVR) learning environments due to the levels of technological immersion and interaction and that (b) higher motor involvement during training (through the total number of object manipulation) has a positive effect on performance compared to less motor involvement.

## MATERIALS AND METHODS

### Participants

Forty-seven participants (28 female and 19 male) were recruited for this study through the digital recruitment platform Accindi (https://www.accindi.se/) with a median age of 24 years (range = 19–40) (Table 1). All participants were right-handed; above 18 years of age; had Swedish as their L1; and had no knowledge of Japanese, Chinese, or any other southeast Asian tonal language. Handedness was assessed by self-reporting their dominant hand for most daily tasks, for example, writing. Further exclusion criteria were any contraindications for ultra-high field MRI, like metal implants, pregnancy, or claustrophobia. All 47 participants completed both MRI examinations and were included in this analysis.

**Table 1.** Differences between all groups within the demographic data are expressed in *p* values.

	No training	dVE	iVR	*p*
*n* (female)	14 (6)	15 (10)	18 (12)	0.31
Age (years)	23.0 (19–34)	25 (21–40)	24.5 (20–39)	0.02
Hours between pre- and post-MRI	2.7 (2.3–3.4)	2.8 (2.2–4.0)	3.0 (2.4–4.3)	0.36
Days between recognition tests	—	6 (2–13)	7 (3–17)	0.17
SART (total correct)	0.91 (0.04)	0.90 (0.04)	0.90 (0.03)	0.64
3-back (*M*)	0.65 (0.23–0.85)	0.72 (0.51–0.83)	0.71 (0.56–0.90)	0.22
4-back (*M*)	0.55 (0.18–0.83)	0.65 (0.47–0.81)	0.69 (0.58–0.91)	0.03

*Note*. Normally distributed data are expressed as mean (standard deviation) and non-normally distributed data as median (range). None of the *p* values survived false discovery rate correction. More detailed statistical information can be found in Supporting Information Table S1, available at https://doi.org/10.1162/NOL.a.238. dVE = desktop-based virtual environment; iVR = immersive virtual reality; MRI = magnetic resonance imaging; SART = Sustained Attention to Response Task.

### Procedure

**Figure 1.**
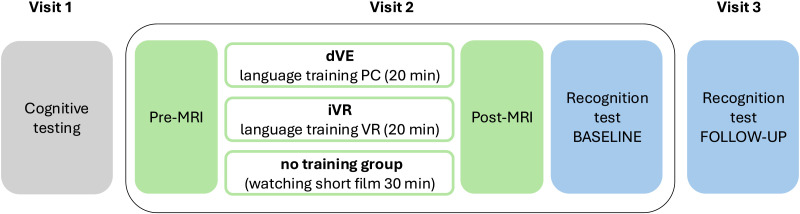
Overview of the study setup. At the first visit, all participants underwent cognitive testing. At the second visit, the dVE and iVR were trained for 20 min in the virtual kitchen, while the no training group watched a short film. All participants underwent pre- and post-intervention MRI scans at the second visit. The dVE and iVR groups performed a baseline recognition test at the end of the second visit and a follow-up of the recognition test approximately 1 week later. dVE = desktop-based virtual environment; iVR = immersive virtual reality; MRI = magnetic resonance imaging.

The data collection was divided across three visits (Figure 1). During the first visit, all participants were tested on the Sustained Attention to Response Task (SART) (Robertson et al., 1997) as well as a 3-back and 4-back working memory tasks to control for possible variations in attention and cognitive control. All cognitive tests were presented and executed within Psychopy 3.2.4 (Peirce et al., 2019). Due to our short training session of only 20 min, it was important for the participants to sustain attention and to focus on relevant information during training. It has previously been demonstrated that high-immersive learning environments might lead to cognitive overload and a lower working memory, and that might be a disadvantage for performance (Lan et al., 2015; Makransky & Petersen, 2021).

**Figure 2.**
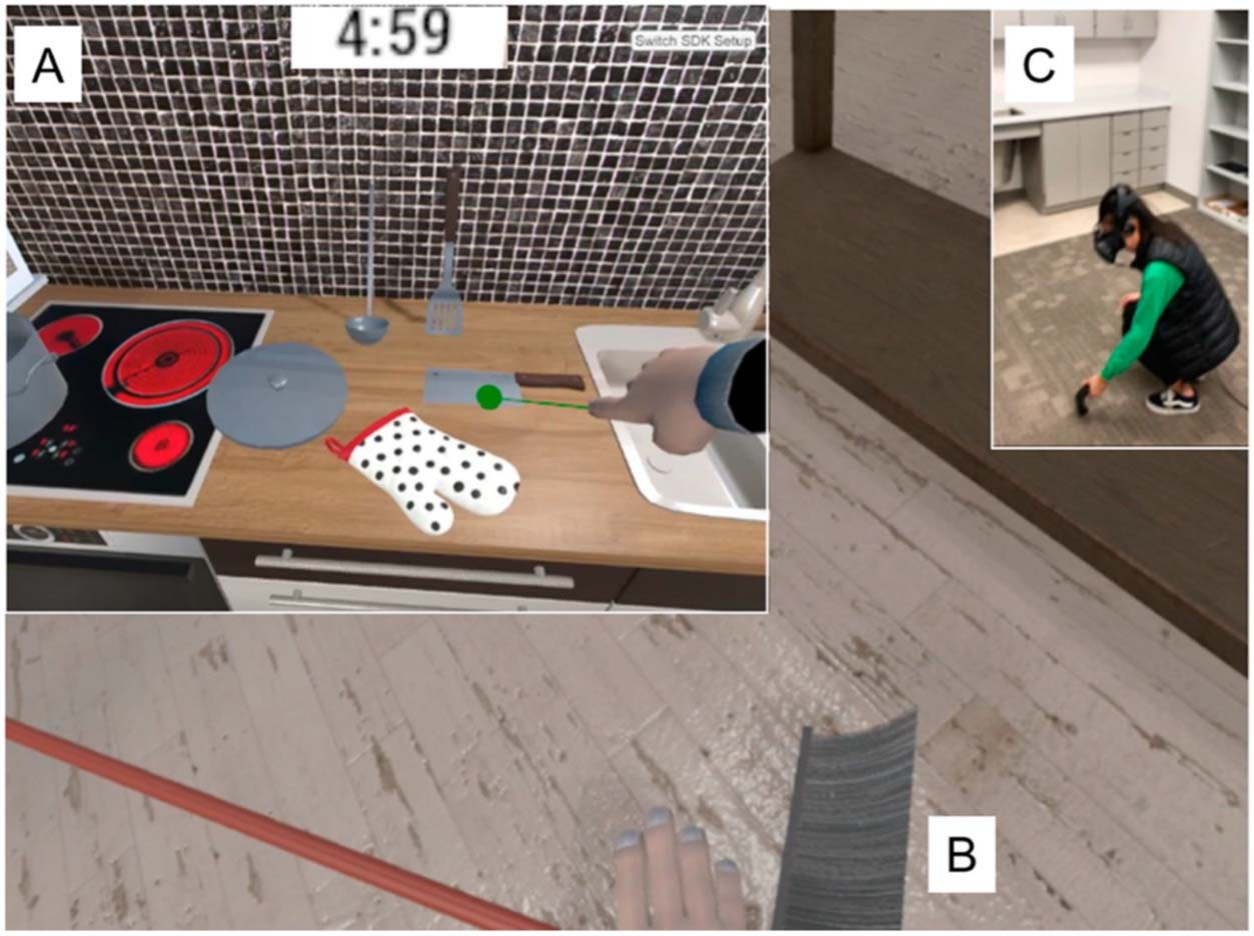
Virtual learning environment (adapted from Legault, Zhao, et al., 2019). (A) By pointing at an object, in this case “dao” (knife in Mandarin Chinese), the name of the object was played. Participants used either the right mouse button (dVE) or the index button at the hand controllers (iVR). (B) Manipulation of an object, a broom, using the left mouse button (dVE) or the grip button at the hand controllers (iVR). (C) Individual in the iVR group picks up an item in panel B. dVE = desktop-based virtual environment; iVR = immersive virtual reality.

At the second visit, which was the main training day, the participants were randomly split into three groups: a low-embodied dVE group using a computer screen, a high-embodied iVR group using an HMD, and a no training group. In the no training condition, participants watched a video in Swedish about hiking and camping in the forest for 30 min to approximately match the time of the training groups plus preparations. The task for the two embodied conditions was to memorize the names of 30 common kitchen objects in Mandarin Chinese over 20 min in a virtual kitchen. The virtual kitchen was the same for both learning groups (Figure 2). The environment was originally developed by Lan et al. (2015) and Legault, Zhao, et al. (2019). All participants underwent pre- and post-intervention MRI scans on the same day, as well as a recognition test, assessing the learned words in both learning groups after the post-MRI (see below).

At the third visit, participants in both training groups were asked to do a follow-up recognition test to examine possible resilient effects.

### Learning Environment

In the iVR group, participants were trained using a Meta Quest 2 HMD at the Humanities Lab at Lund University. They started with a practice round and were instructed on how to point and manipulate objects with hand controllers and how to move around within the virtual boundaries in first-person view. After the practice round, they were instructed again about the aim of the task and were informed about the time limit of 20 min. During the learning task, the experimenter observed the participants by casting the iVR view on a computer screen.

In the dVE group, the participants were trained via the same virtual kitchen but on a computer screen in the Humanities lab. Like the iVR condition, they also started with a short practice condition. They were instructed to use the right mouse button to point at objects and to use the left mouse button to manipulate objects. It was possible to move around in the environment using the arrow keys on the keyboard.

In both conditions, when the participants pointed at an object, the name of the object in Mandarin Chinese was played. Every 5 min, objects that were not yet pointed at by the participants were indicated by a red arrow to guarantee that all objects were learned. In the virtual kitchen, it was also possible to manipulate and interact with the virtual objects (grab, rotate, or move). This was done either by real hand movements in the iVR group or using the mouse in the dVE group. When the participants manipulated an object, the object name was not played.

### Behavioral Data

The following behavioral data were collected during the task: (a) how often participants pointed at each object, (b) how often they manipulated each object, and (c) the distance in meters they moved or walked around in the virtual kitchen. The movement inside the virtual kitchen was recorded in the horizontal plane. In the iVR condition, it was real walking compared to dVE where participants moved around in first-person view in the kitchen with the arrow keys on a keyboard.

### Recognition Test

Participants in the learning groups were tested on all 30 words learned after the post-MRI scan (baseline) at the second visit and 1 week later (follow-up) at the third visit. During the recognition test, three images were presented in the middle of a computer screen using Psychopy 3.2.4 (Peirce et al., 2019). After 1.5 s, the name of the object was presented auditorily once, and the participants were instructed to choose the correct image corresponding to the object by pressing 1, 2, or 3 on the computer keyboard. To test whether the learned object names were associated with the virtual or with real objects, participants were tested on each object twice using the virtual, rendered image and a real, generic photography in a random order.

To summarize, we used three different performance measures for each recognition test: (a) the proportion of correct answers for the rendered images, (b) the proportion of correct answers for the real images, and (c) an accumulated performance (total) based on the two previous tests by including rendered as well as real images.

### MRI Acquisition

All participants underwent two MRI scans on the training day, before and after the learning task, using an actively shielded Philips Achieva 7T MRI scanner (Philips, Best, The Netherlands) equipped with an eight-channel transmit and a 32-channel receive head coil array (Nova Medical, Wilmington, MA). Compared to a 3T MRI, the 7T MRI has higher signal-to-noise ratio, which allows higher resolution. This might be beneficial to detect subtle and rapid gray matter changes due to our short training session. To reduce radiofrequency (B1) inhomogeneities, dialectic pads were used between the head coil and the occipitotemporal region of the head for each participant (Teeuwisse et al., 2012). The sequences included in this study were a T1-weighted three-dimensional magnetization-prepared rapid gradient echo sequence (Mugler & Brookeman, 1990) (voxel size = 0.7 × 0.7 × 0.7 mm^3^, repetition time (TR)/echo time (TE) = 8/2.6 ms, inversion time (IT) = 1,200 ms, turbo field echo (TFE) shots = 149, TFE shot length = 2,000 ms, flip angle = 7°, inplane SENSE factor = 1.4, spacing between slices = 0.35 mm using overcontiguous slices) and, at the first MRI, a fluid-attenuated inversion recovery 3D scan (voxel size = 2.4 × 0.7 × 0.7 mm, TR/TE/TI = 6,000/284/1,825 ms, flip angle = 55°, inplane SENSE factor = 2) to control for possible lesions or brain abnormalities.

### VBM

The T1-weighted images were reoriented to MNI152 standard template orientation using *fslreorient2std* in FMRIB Software Library (FSL) software 6.0.6 (Jenkinson et al., 2012) and bias field corrected using N4 in Advanced Normalization Tools 2.4.2 (Tustison et al., 2010). VBM pre-processing was performed using FSL-VBM software 6.0.6. (Jenkinson et al., 2012; Smith et al., 2004). In short, skull-stripping was performed on all images using adapted parameters (fractional intensity threshold = 0.1, vertical gradient = 0.1). A manual quality assessment was performed, and the skull-stripping was manually improved for seven MRI images by changing the brain extraction parameters. Next, a study-specific template was created using the pre-intervention MRI of 42 participants. Participants included in the template were balanced across all three groups (14 from each group). In detail, all 14 controls and 14 randomly chosen participants from the dVE as well as iVR group were included. Following this, all images were non-linearly registered to the study-specific template. All images were smoothed using an 8-mm full width at half maximum (FWHM) Gaussian kernel. The size of the smoothing kernel was chosen to reduce the effect of misregistrations without losing spatial sensitivity (Jones et al., 2005; Smith & Nichols, 2009). Additionally, to test the robustness of our results, we ran the analysis with four additional smoothing kernels: 2, 4, 6, and 10 mm FWHM, which allowed us to reproduce our main findings also with a filter size of 4 and 6 mm.

### Statistical Analysis

The pre-processed neuroimaging data were analyzed by means of FSL *randomise* (Winkler et al., 2014) for group comparisons. We calculated the difference between the modulated gray matter images for each participant (post-MRI minus pre-MRI) and corrected each analysis for age, sex, and total intracranial volume (TIV) (Barnes et al., 2010). The TIV was estimated by the brain masks from the pre-intervention MRI.

We restricted the gray matter analysis to our regions of interest (ROIs) based on the above-mentioned literature: the bilateral IPL, the bilateral IFG, as well as the bilateral superior temporal gyrus, a region mainly involved in acoustic and phonological processing especially for tone processing in Chinese (Li et al., 2014). We also included the bilateral middle temporal gyrus (MTG) as well as the bilateral hippocampus, two vital parts in lexical–semantic associations and processing (Li & Grant, 2016; Li et al., 2014). We created a combined binary mask, which includes all above-mentioned regions. The mask was exported from the Harvard–Oxford cortical structural brain atlas, 2 mm with a threshold of zero (Desikan et al., 2006; Frazier et al., 2005; Goldstein et al., 2007; Makris et al., 2006). Additionally, we performed an exploratory whole-brain analysis to reveal possible memory or learning-related effects outside of our ROIs. If not stated differently, threshold-free cluster enhancement (TFCE) method implemented in FSL was applied on the combined ROI mask as well as over the whole-brain gray matter mask to correct for multiple comparisons (Smith & Nichols, 2009).

To explore the effect of object manipulation, we performed a linear regression model between the total number of manipulations and performance measures. Additionally, we split the participants in both learning groups into a “high-manipulator” and a “low-manipulator” group using a median split analysis based on the total number of object manipulations (median = 10).

To examine if there is a difference between performance groups, we split the participants into low- and high-performer based on the recognition test at baseline using the total score (median = 0.92). Group differences for nominal variables were assessed with chi-square tests and Fisher’s exact tests for pairwise comparisons. For continuous variables, a one-way analysis of variance (ANOVA) for parametric distributed data and a Kruskal–Wallis test for non-parametric distributed data were used. Post hoc comparisons were conducted with Tukey post hoc and Dunn tests. Differences between two groups were performed using a *t* test for parametric as well as a Mann–Whitney *U* test for non-parametric distributed data. False discovery rate corrections were performed in Python using *statsmodel 0.14.0* (Seabold & Perktold, 2010). Tests on data normality (Shapiro–Wilk test) as well as all other statistical tests were performed in Python using *Scipy 1.11.3* (Virtanen et al., 2020).

## RESULTS

### The Effect of Learning Setup

The age in the dVE group (*p* = 0.036, *t*-statistics = 2.42, effect size Cohen’s *d* = 0.97) as well as in both training groups combined (*p* = 0.047, *t*-statistics = 2.04, effect size Cohen’s *d* = 0.66) was significantly higher compared to the no training group. Additionally, participants in the iVR group performed higher in the 4-back compared to the no training group (*p* = 0.006, *t*-statistics = 3.11, effect size Cohen’s *d* = 1.04). No difference was found between groups in sustained attention (SART) performance (Table 1).

When comparing the training groups, there was no significant difference in horizontal movement or in the number of times the participants pointed or manipulated an object between the dVE and iVR groups (Table 2). Furthermore, there were no differences in baseline and follow-up performance measures between the dVE and the iVR groups. However, there was a tendency in the dVE group for slightly better learning outcomes than in the iVR condition, but this was not statistically significant. Additionally, we could not find any difference between the low- and high-performer groups in the cognitive test scores nor in the behavioral measures.

**Table 2.** Behavioral data.

	dVE	iVR	*p*
**Behavioral data**			
Number of object-pointings	481 (130)	475 (135)	0.89
Number of object manipulations	5 (0–89)	12 (0–113)	0.73
Horizontal movement in meters^a^	89 (94)	132 (37)	0.08
**Recognition test baseline**			
Total	0.93 (0.81–1.0)	0.87 (0.56–1.0)	0.12
Real images	0.93 (0.73–1.0)	0.88 (0.6–1.0)	0.18
Rendered images	0.97 (0.83–1.0)	0.91 (0.53–1.0)	0.12
**Recognition test follow-up** ^b^			
Total	0.92 (0.74–0.98)	0.86 (0.58–0.98)	0.12
Real images	0.93 (0.70–1.0)	0.90 (0.60–0.97)	0.10
Rendered images	0.90 (0.77–1.0)	0.83 (0.57–1.0)	0.22

*Note*. Differences between the dVE and iVR groups for behavioral and performance values are expressed in *p* values. Normally distributed data are expressed as mean (standard deviation) and non-normally distributed data as median (range). None of the *p* values survived false discovery rate correction. More detailed statistical information can be found in Supporting Information Table S2. dVE = desktop-based virtual environment; iVR = immersive virtual reality.

^a^ Movement in iVR was real movement, whereas the dVE group moved with mouse and keyboard arrow keys.

^b^ One subject in the iVR condition missed the recognition test at follow-up.

**Figure 3.**
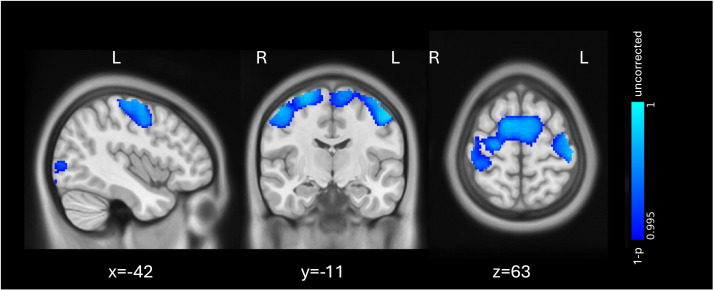
Higher gray matter volume in dVE compared to iVR using a whole-brain analysis with a *p* value of < 0.005 without TFCE correction. Blue-cyan: uncorrected results (*p* < 0.005). The results are shown in MNI space and adjusted for sex, age, and TIV. dVE = desktop-based virtual environment; iVR = immersive virtual reality; MNI = Montreal Neurological Institute template; TIV = total intracranial volume; TFCE = threshold-free cluster enhancement.

Changes in the gray matter volume in our ROIs and whole-brain analysis between the no training group and the training groups combined, as well as between the dVE and iVR groups, were not significant after TFCE correction. However, without TFCE correction, the dVE showed higher gray matter volume in the bilateral primary sensorimotor cortex, supplementary motor area (SMA), as well as the left lateral occipital cortex (LOC), compared to iVR (Figure 3).

### The Effect of Object Manipulation

To examine the effect of object manipulation, we performed a linear regression between the total number of manipulations and performance measures. Results showed a negative trend for recognition test baseline total (*R*^2^ = 0.037, *p* value = 0.27, 95% confidence intervals slope = −0.0017, 0.0005) and recognition test follow-up total (*R*^2^ = 0.088, *p* value = 0.09, 95% confidence intervals slope = −0.002, 0.002), but no statistical significance. To further examine the impact of manipulation, we divided participants into low- and high-manipulators using a median split based on the total number of manipulations over all participants within both learning groups (Table 3). The median manipulations were three (range 0–10) for low-manipulators and 34 (range 13–113) for high-manipulators, resulting in a significant difference between the two groups (*p* < 0.001), as expected due to the median split that was performed earlier. Low-manipulators demonstrated significantly higher performance at both baseline and follow-up sessions compared to high-manipulators, except for the performance measure at baseline using rendered images (Table 3).

**Table 3.** Comparison between low- versus high-manipulators.

	Low-manipulator	High-manipulator	*p*
Ratio dVE/iVR	8/9	7/9	—
*n* (female)	17 (13)	16 (9)	0.28
Age (years)	24 (22–40)	25 (20–39)	0.65
3-back (*M*)	0.69 (0.07)	0.72 (0.11)	0.36
4-back (*M*)	0.68 (0.10)	0.68 (0.10)	0.98
SART (total correct)	0.89 (0.04)	0.91 (0.03)	0.34
**Behavioral data**			
Number of object-pointings	477 (147)	479 (116)	0.95
Number of object manipulations	3 (0–10)	34 (13–113)	**< 0.001**
Horizontal movement in meters^a^	93 (60)	134 (78)	0.10
**Recognition test baseline**			
Total	0.95 (0.76–1.0)	0.88 (0.56–0.98)	**0.016**
Real images	0.93 (0.80–1.0)	0.87 (0.60–1.0)	**0.004**
Rendered images	0.97 (0.73–1.0)	0.90 (0.53–1.0)	0.073
**Recognition test follow-up** ^b^			
Total	0.92 (0.84–0.98)	0.80 (0.58–0.98)	**0.004**
Real images	0.93 (0.77–1.0)	0.83 (0.57–0.97)	**0.011**
Rendered images	0.93 (0.77–1.0)	0.80 (0.60–1.0)	**0.019**

*Note.* The low-manipulators showed significantly higher performance compared to the high-manipulators, except for the performance measure at baseline using rendered images. **Bold** *p* values indicate false discovery rate corrections. More detailed statistical information can be found in Supporting Information Table S3. dVE = desktop-based virtual environment; iVR = immersive virtual reality.

^a^ Movement in iVR was real movement, whereas the dVE group moved with mouse and keyboard arrow keys.

^b^ One subject in the iVR condition had to be removed due to missing follow-up data.

**Figure 4.**
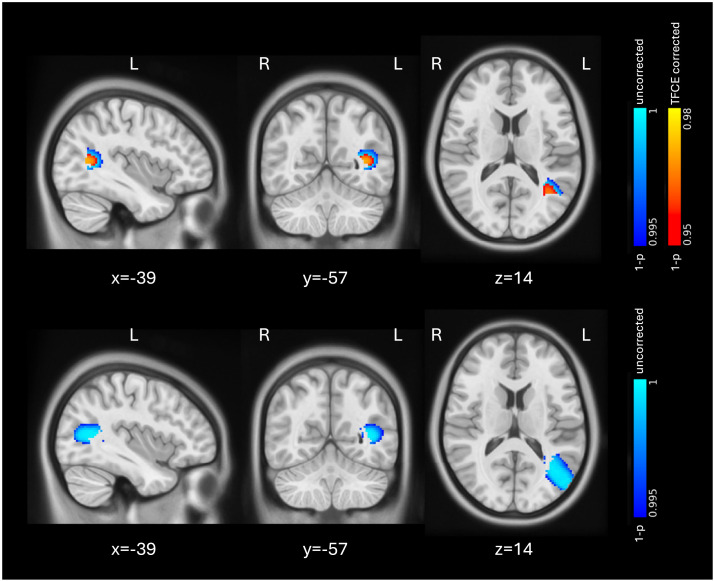
Higher gray matter volume in low-manipulators compared to high-manipulators. Top: Changes in our region of interest. Bottom: Whole-brain analysis. Red-yellow: significant voxels after TFCE correction (*p* < 0.05). Blue-cyan: uncorrected results (*p* < 0.005). The results are shown in MNI space and adjusted for sex, age, and TIV. MNI = Montreal Neurological Institute template; TIV = total intracranial volume; TFCE = threshold-free cluster enhancement.

When comparing gray matter volume changes between the low-manipulators and high-manipulator, we found a significant higher gray matter volume in the low-manipulators compared to the high-manipulators in the left IPL, in particular in the left angular gyrus as well as in the left MTG within our ROIs (Figure 4, top). The whole-brain analysis showed higher gray matter volume in the left LOC in the low-manipulators compared to the high-manipulators without TFCE correction (Figure 4, bottom).

## DISCUSSION

In this study, we investigated the effect of technical immersion and sensorimotor engagement on learning outcome and rapid structural brain changes after a short language learning task. Our first hypothesis regarding structural brain differences between the low- and high-embodied learning environments was not confirmed by our data and analyses. We did not find any differences in gray matter volume between the dVE and iVR groups, nor between the learning groups combined compared to the no training group in our ROIs.

There are several possible reasons for this. As mentioned above, one longitudinal study that used the same dVE learning environment identified the right IPL as well as the right IFG as possible context-dependent structures within the language control network (Legault, Fang, et al., 2019). However, the authors compared this to a non-embodied 2D picture–word association on a computer screen and the participants trained over several sessions. Although there are clear differences between dVE and iVR, in particular the level of technological immersion and body–object interactions, both learning setups might rely on similar cortical structures. The inclusion of a third non-VR learning group, with no immersion or interactions (e.g., paper-based picture–word learning), might have revealed a more comprehensive picture of immersion and sensorimotor engagement and potential associations with brain plasticity.

The exploratory whole-brain analysis showed higher gray matter volume in the primary sensorimotor cortex as well as the SMA in the dVE group compared to the iVR group (Figure 3). Although this effect is uncorrected, we argue that there is a learning effect on how participants in the dVE group learned to navigate with the computer mouse and keyboard in the virtual environment, compared to the more natural body movements in the iVR group. We can exclude an effect of object manipulation because we could not find the same areas in the whole-brain comparison between low- and high-manipulators.

A lack of significant training might be another reason why we could not find differences between the embodied learning groups, and with no observed effect of training on the brain when both training groups were compared to the no training group. A longer training exposure, for example, with more words, multiple sessions over several days or weeks, might have revealed training-related changes within our ROI. However, the focus of this study design was on rapid structural changes. Interestingly, with only 20 min of training, our data showed that performance measures were very high, which resulted in a ceiling effect. A task in which more words were included, for example, 40 instead of 30 words in 20 min, would have reduced the ceiling effect and potentially allowed us to distinguish between high- and low-performers.

In summary, although our learning task was highly embodied, the high immersive and interactive learning stimulus did not cause detectable structural changes using VBM, and we could not find differences between the two different learning groups.

Contrary to our second hypothesis, participants with fewer object manipulations showed significantly better performance at baseline and follow-up sessions compared to those with a higher number of manipulations. Low-manipulators showed higher gray matter volume in an area related to the left angular gyrus and the temporooccipital part of the left MTG compared to high-manipulators (Figure 4, top). This seems to be a performance-dependent effect on the left angular gyrus, as part of the left IPL, which is in line with previous research. According to a number of studies, the left IPL reflects performance and learning efficiency in general L2 learning (Li & Lan, 2022; Li et al., 2020; Mechelli et al., 2004). A similar study using the same dVE found an increase in cortical thickness in the left IPL, as well as a positive correlation of gray matter volume with performance, also in the left IPL (Legault, Fang, et al., 2019).

Another area that showed a manipulation-dependent effect was the left MTG. The left MTG is a central hub within the language control network (Li & Grant, 2016) and plays a role in lexical and conceptual semantic processing (Grant et al., 2015; Legault, Grant, et al., 2019; Li & Jeong, 2020). The difference in the MTG might be related to performance, which could mean that a higher number of manipulations was a disadvantage for the semantic processing of the virtual objects.

However, to strengthen our conclusion, it would have been necessary to include a non-embodied learning group as well as to increase the number of participants. Even though compared to similar studies examining the effect of VR technology on behavior and the brain, our overall group size is higher, it still remains modest for each condition examined (Button et al., 2013; Kharabian Masouleh et al., 2019). Additionally, apparent tissue changes can also be caused by daily variation in intake such as caffeine consumption (Ge et al., 2017), which also limits the interpretation of our findings.

To summarize our findings on sensorimotor engagement, in our setup it seems that less sensorimotor involvement during learning has a positive impact on performance and causes changes in the left IPL as well as the left MTG compared to more sensorimotor involvement.

Interestingly, our uncorrected whole-brain analyses revealed that the left LOC, which is an area known to be involved in object recognition and perception (Grill-Spector, 2003; Grill-Spector et al., 2001), showed higher gray matter volume in dVE compared to iVR (Figure 3) and low-manipulators compared to high-manipulators (Figure 4, bottom). Based on these findings, we hypothesize that the full embodied 3D environment and the higher number of manipulations might have had a negative effect on object recognition and further performance. In an fMRI study on auditory L2 vocabulary training, learning with 2D pictures showed an activation in the right LOC compared to gestures (Mayer et al., 2015). Although our findings in the left LOC seem to be in line with the literature, our results are uncorrected and do not survive multiple comparison.

Why do our 3D environment (iVR) and the higher number of object manipulations have a negative impact on performance and object recognition? There are several reasons that might explain such an effect. One explanation could be that the expectations from the participants on object interaction on iVR were not met within our task. Recent studies have shown that when participants’ expectations in iVR on agency control of the virtual content are not met, a strong negative effect on learning may occur (Johnson-Glenberg et al., 2021). Another reason could be related to movement congruency. Congruency can be defined as an overlap between the performed actions and the learning content, which is defined as the third main dimension in VR-enhanced learning, beside immersion and interaction (Johnson-Glenberg, 2018). According to the literature, there are clear benefits from motor actions during learning using VR technology (Johnson-Glenberg et al., 2014). However, the type of manipulation or action and gesture plays an important role. In a study on object manipulation using VR technology, participants who performed a manipulation movement that implied the objects’ usage (relevant movement) performed significantly better compared to those performing irrelevant movements (Fuhrman et al., 2021). The authors suggested that congruent movements add enriched information to the mnemonic process when learning object names in a new language. A similar effect was found using co-speech gestures (Kelly et al., 2009), which might indicate that non-matching gestures with the learning content may be more hindering than learning without gestures (Gullberg, 2024). However, a recent electroencephalography (EEG) study that investigated action congruency using VR technology found no semantic or motor effect when comparing to action observing (Zappa et al., 2024).

Based on previous literature, we believe that in our study, the high degree of freedom in the virtual environment and in manipulating objects was more distractive than advantageous (Makransky et al., 2019). This effect is unlikely to be related to attention or working memory because there were no differences in the SART nor in the *n*-back tasks between the low- and high-manipulators (Table 3). The high-manipulators in our study presumably had higher sensorimotor engagement, but their manipulations on the objects might have lacked congruency and did not mimic any functional or relevant meaning. Participants turned over or moved the virtual kitchen objects, like spoons, cups, or teapots, but not in a manner that closely resembled actual everyday use. It is worth mentioning that the time for manipulations did not restrict the time spent pointing at objects. However, as shown by Zappa et al. (2024), the missing action congruency in our study when manipulating objects might not fully explain the disadvantage of higher object manipulations.

Another reason could be that different performance groups use different learning strategies. A study on L2 vocabulary training in the same virtual kitchen using dVE has shown that low- and high-performers use different learning strategies (Hsiao et al., 2017). In a similar study, less successful learners benefit more from higher immersion and sensorimotor engagement compared to more successful learners (Legault, Zhao, et al., 2019). It is possible that the less successful learners in our experiment tried to compensate for their disadvantage by performing more manipulations.

The advantages of iVR compared to dVE learning seem to be obvious: higher level of immersion, whole-body movement, and more vivid and inclusive experience. However, we could not find any differences between the two learning groups in performance. Our results are in line with the current literature, which does not point toward a clear superiority of dVE or iVR for L2 learning (Li et al., 2020). In Li et al. (2020), the authors argue that individual differences, for example, interaction with VR features, play a more important role in performance than the level of immersion. In our study, we came to the same conclusion, but with the opposite effect of interaction with virtual objects (i.e., the less the better). Previous studies that have used the same learning environments compared both the low-embodied dVE (Lan et al., 2015) as well as the high-embodied iVR learning environment (Legault, Zhao, et al., 2019) to a non-immersive learning setup, and both showed a superiority toward dVE and iVR, respectively. In our direct comparison of dVE and iVR, we could not find any gray matter volume differences or association of the bilateral IPL with immersive learning or embodied cognition. As already mentioned above, a reason for this could be the study design or the low number of participants within each group. However, we could show, in a short-term learning setting, that object manipulation plays a more important role than the level of immersion in performance and learning outcome.

## CONCLUSION

Neuroimaging studies using immersive and interactive L2 learning environments have indicated that cortical structures within the language control network are dependent on the learning context and in particular in technological immersion. Our study, with training over a single session, could not confirm such differences, which might indicate that both learning environments rely on similar cortical structures, or that these changes only occur following longer periods of training. Participants with lower object manipulations performed significantly better at baseline and follow-up, and these performance differences were also associated with higher gray matter volume in the left angular gyrus and left MTG, compared to high object manipulation. There are a few directions future studies can pursue in this line of work. Although our analysis yielded statistically significant effects in the ROI, we must be careful when drawing broader conclusions about the effect of object manipulation on performance and potential brain changes due to the low number of participants included as well as due to our short vocabulary training.

To summarize, a new technology does not always improve learning, and the design of such environments, for example, how to implement and control for sensorimotor engagement and object recognition of virtual content, has a strong impact on the learning outcome in VR-supported learning.

## Acknowledgments

We would like to thank all participants who took part in this study as well as Sara Farshchi for proofreading the manuscript.

## Funding Information

Johan Mårtensson, Vetenskapsrådet (https://dx.doi.org/10.13039/501100004359), Award ID: 2017-00896. Johan Mårtensson, Marcus och Amalia Wallenbergs minnesfond (https://dx.doi.org/10.13039/501100011898). Ping Li, Research Grants Council, University Grants Committee (https://dx.doi.org/10.13039/501100002920), Award ID: PolyU15610322. Ping Li, Sin Wai Kin Foundation.

## Author Contributions

**Theodor Rumetshofer**: Conceptualization; Data curation; Formal analysis; Investigation; Methodology; Project administration; Resources; Validation; Writing – original draft, Writing – review and editing. **Lara Langensee**: Conceptualization; Data curation; Software; Methodology; Project administration; Writing – review and editing. **Ping Li**: Conceptualization; Funding acquisition; Methodology; Software; Supervision; Writing – review and editing. **Jiayan Zhao**: Resources; Software; Writing – review and editing. **Alexander Klippel**: Software; Writing – review and editing. **Linda Wennberg**: Investigation; Methodology; Project administration; Resources; Writing – review and editing. **Markus Nilsson**: Methodology; Resources; Validation; Writing – review and editing. **Pia C. Sundgren**: Funding acquisition; Resources; Validation; Writing – review and editing. **Marianne Gullberg**: Conceptualization; Funding acquisition; Methodology; Supervision; Writing – review and editing. **Johan Mårtensson**: Conceptualization; Funding acquisition; Investigation; Methodology; Project administration; Resources; Supervision; Validation; Visualization; Writing – original draft; Writing – review and editing.

## Code and Data Availability Statements

The statistical maps as well as the behavioral data of this study are publicly available at https://github.com/TheoRum/manuscript_embodied_learning_VBM. The analysis on the stuructural MRI data was performed using the open-source software FSL-VBM (Version 6.0.6; https://web.mit.edu/fsl_v5.0.10/fsl/doc/wiki/FSLVBM.html).

## Ethics Statement

The Swedish Ethical Review Authority approved the study (#2019-05387), and written informed consent was obtained for all participants prior to inclusion. The study was in accordance with the relevant guidelines and regulations from the Declaration of Helsinki.

## Supplementary Material



## TECHNICAL TERMS

Voxel-based morphometry (VBM): A volumetric analysis that allows a voxel-wise investigation of T1-weighted images from magnetic resonance imaging (MRI).
Threshold-free cluster enhancement (TFCE): A voxel-wise statistical method to enhance areas of signal with a certain spatial connection.
