# Supplementary material for: Impact of Technological Immersion and Sensorimotor Engagement on Performance and Brain Plasticity in Short-Term Second Language Vocabulary Training
Source: Neurobiol Lang (Camb). 2026 Mar 27;7:NOL.a.238. doi: 10.1162/NOL.a.238 (PMC13065095; doi:10.1162/NOL.a.238)
Supplement: Supplementary file 1 [file nol-07-238-s001.pdf]

Supplementary information

Table S1: An extended version of table 1 which includes the appropriate statistics. Differences between all groups within the demographic data are expressed in *p*-values. Normally distributed data are expressed as mean (standard deviation) and non-normally distributed data as median (range). None of the *p*-values survived False Discovery Rate (FDR) correction. The null-hypothesis from the Shapiro-Wilks test was that the data were drawn from a normal distribution.

SART = Sustained attention to response task, dVE = desktop-based virtual environment, iVR = immersive virtual reality

	**no training**	**dVE**	**iVR**	***p*-value**	**statistics**	**Shapiro-Wilk *p*-value**
n (female)	14 (6)	15 (10)	18 (12)	0.31	X^2=2.3	>0.001
age (years)	23.0 (19-34)	25 (21-40)	24.5 (20-39)	0.02	H=7.7	>0.001
hours between pre- and post MRI	2.7 (2.3-3.4)	2.8 (2.2-4.0)	3.0 (2.4-4.3)	0.36	H=2.0	0.014
days between recognition tests	-	6 (2-13)	7 (3-17)	0.17	U=91.5	>0.001
SART (total correct)	0.91 (0.04)	0.90 (0.04)	0.90 (0.03)	0.64	F=0.43	0.133
3-back (mean)	0.65 (0.23-0.85)	0.72 (0.51-0.83)	0.71 (0.56-0.90)	0.22	H=3.0	0.003
4-back (mean)	0.55 (0.18-0.83)	0.65 (0.47-0.81)	0.69 (0.58-0.91)	0.03	H=6.5	0.007

Table S2: An extended version of table 2 which includes the appropriate statistics. Differences between the dVE and iVR groups for the behavioural and performance data are expressed in *p*-values. Normally distributed data are expressed as mean (standard deviation) and non-normally distributed data as median (range). None of the *p*-values survived False Discovery Rate (FDR) correction. The null-hypothesis from the Shapiro-Wilks test was that the data were drawn from a normal distribution.

†Movement in iVR was whole body movement whereas the dVE group moved with mouse and keyboard arrow keys.

*One subject in the iVR condition missed the recognition test at follow-up.

dVE = desktop-based virtual environment, iVR = immersive virtual reality

	**dVE**	**iVR**	***p*-value**	**statistics**	**Effect size (Cohens'd)**	**Shapiro-Wilk *p*-value**
**Behavioural data**						
number of object-pointings	481 (130)	475 (135)	0.89	T=0.12	0.04	0.32
number of object manipulations	5 (0-89)	12 (0-113)	0.73	U=145.0	>0.01	>0.001
horizontal movement in meters †	89 (94)	132 (37)	0.08	T=1.78	0.64	0.15
**Recognition test baseline**						
total	0.93 (0.81-1.0)	0.87 (0.56-1.0)	0.12	U=92.0	0.66	>0.001
real images	0.93 (0.73-1.0)	0.88 (0.6-1.0)	0.18	U=98.0	0.57	0.01
rendered images	0.97 (0.83-1.0)	0.91 (0.53-1.0)	0.12	U=92.5	0.70	>0.001
**Recognition test follow-up***						
total	0.92 (0.74-0.98)	0.86 (0.58-0.98)	0.12	U=86.5	0.62	0.01
real images	0.93 (0.70-1.0)	0.90 (0.60-0.97)	0.10	U=85.0	0.67	0.03
rendered images	0.90 (0.77-1.0)	0.83 (0.57-1.0)	0.22	U=95.5	0.42	0.004

Table S3: An extended version of table 3 which includes the appropriate statistics. Comparison between low- versus high-manipulators. The low-manipulators showed significant higher performance compared to the high-manipulators expect for rendered images at baseline. Bold *p*-values indicate False Discovery Rate (FDR) corrections. The null-hypothesis from the Shapiro-Wilk was that the data were drawn from a normal distribution.

*one subject in the iVR condition had to be removed due to missing follow-up data.

dVE = desktop-based virtual environment, iVR = immersive virtual reality.

	**low-manipulator**	**high-manipulator**	***p*-value**	**statistics**	**Effect size (Cohens'd)**	**Shapiro-Wilk *p*-value**
ratio dVE / iVR	8 / 9	7 / 9	-	-	-	-
n (female)	17 (13)	16 (9)	0.28	-	-	>0.001
age (years)	24 (22-40)	25 (20-39)	0.65	U=123.0	0.36	>0.001
3-back (mean)	0.69 (0.07)	0.72 (0.11)	0.36	T=0.91	0.33	0.75
4-back (mean)	0.68 (0.10)	0.68 (0.10)	0.98	T=0.01	>0.01	0.46
SART (total correct)	0.89 (0.04)	0.91 (0.03)	0.34	T=0.95	0.34	0.49
**Behavioural data**						
number of object-pointings	477 (147)	479 (116)	0.95	T=0.05	0.01	0.32
number of object manipulations	3 (0-10)	34 (13-113)	**<0.001**	U=272.0	1.90	>0.001
horizontal movement in meters †	93 (60)	134 (78)	0.10	T=1.67	0.60	0.15
**Recognition test baseline**						
total	0.95 (0.76-1.0)	0.88 (0.56-0.98)	**0.016**	U=69.0	0.84	>0.001
real images	0.93 (0.80-1.0)	0.87 (0.60-1.0)	**0.004**	U=58.0	1.10	0.01
rendered images	0.97 (0.73-1.0)	0.90 (0.53-1.0)	0.073	U=86.5	0.54	>0.001
**Recognition test follow-up***						

total	0.92 (0.84-0.98)	0.80 (0.58-0.98)	**0.004**	U=52.0	1.24	0.01
real images	0.93 (0.77-1.0)	0.83 (0.57-0.97)	**0.011**	U=61.0	1.08	0.03
rendered images	0.93 (0.77-1.0)	0.80 (0.60-1.0)	**0.019**	U=66	1.12	0.004
